# Can major public health emergencies increase the participation of commercial insurance? Evidence from China

**DOI:** 10.3389/fpubh.2024.1363451

**Published:** 2024-05-23

**Authors:** Yiqiu Wang, Chong Guo, Yang Xu, Meng Xie

**Affiliations:** ^1^School of Finance, Nanjing Agricultural University, Nanjing, China; ^2^School of Economics and Management, Henan Agricultural University, Zhengzhou, China

**Keywords:** COVID-19, commercial insurance, risk, digital finance, difference-in-differences

## Abstract

**Background:**

Public health emergencies have a lasting impact on a country's economic and social development. However, commercial insurance can disperse these negative consequences and reduce risk losses.

**Method:**

Based on the Chinese Household Tracking Survey and Peking University Digital Inclusive Finance Index, this study employed a difference-in-differences model to test the impact of the COVID-19 outbreak on commercial insurance participation and the impact mechanism.

**Results:**

The analysis showed that the outbreak of COVID-19 improved residents' risk perception, risk preference and digital finance and promoted their participation in commercial insurance, commercial endowment insurance, and commercial medical insurance.

**Conclusion:**

Major public health emergencies can increase commercial insurance participation, but the promotional effect of commercial insurance on rural and low-income individuals is relatively limited. To tap into potential customers, financial institutions should focus on vulnerable societal groups. This study supplements the relevant literature on the impact of major public health emergencies on commercial insurance participation.

## 1 Introduction

The COVID-19 pandemic was a major public health emergency since the founding of the People's Republic of China, spreading rapidly and proving difficult to prevent and control due to its high level of contagion. It threatened lives and also had a lasting impact on the country's economic and social development. It has exposed shortcomings in the prevention and control system for health emergencies. Insurance is a modern risk-management method that effectively disperses the negative consequences of disasters through the establishment of new types of insurance, the use of reinsurance to disperse risks, and the issuance of catastrophe bonds (1, 2). This is important to a country's emergency system. Commercial insurance is the main supplement to social insurance and has recently become more popular among Chinese households to protect their property and health. However, the participation rate in commercial insurance in China is behind the world average (3) and is still in the early development stage compared to other developed countries (4).

Current literature shows that the limited participation of Chinese in the commercial insurance market is an important factor restricting the development of the commercial insurance industry (5). Research has found that factors such as lack of financial knowledge, weak insurance awareness, and insufficient cognition concerning its purpose have led to a small number of residents purchasing commercial insurance. This, in turn, has led to insufficient insurance demand (3, 6, 7). Risk is a prerequisite and foundation for insurance, and risk awareness is an important condition for generating insurance demand. Risk attitude is a key factor affecting the adoption of family risk management tools. Therefore, families with strong cognitive abilities are more aware of the various risks that they face (8). Scholars have analyzed changes in commercial insurance demand from a macro perspective. For example, Chang and Berdiev (9) examined the relationship between natural disasters, political risk, and insurance market development and found that the incidences of natural disasters and deaths caused by these disasters lead to greater total insurance, life insurance, and non-life insurance consumption. Gallagher (10) and Atreya et al. (11) both found insurance take-up spikes after major floods. Lin (12) explored the relationship between earthquakes and insurance demand and found that insurance demand temporarily increased slightly in areas experiencing moderate-intensity shaking or multiple mild shaking events. With the sudden outbreak of COVID-19 worldwide, isolation measures and intensive exposure information disclosure have led to a significant increase in the attention given to health risks. Whether commercial insurance participation has changed accordingly needs to be investigated further.

COVID-19 has changed the development model of the economy, including the digital economy (13, 14). Digital finance plays an important role in supporting digital economic development and provides a convenient and contactless payment method that reduces shopping costs through a high degree of integration with residents' digital lives (15). The continuous increase in the use of Internet technology in the financial field has improved the accessibility of financial services (16, 17). Scholars discovered that digital finance can significantly positively affect insurance development in terms of improving insurance availability, reducing market transaction costs, and alleviating information asymmetry. Fuster et al. (18) found that with the development of digital finance, it is easier for the public to access risk management education and enhance their risk management awareness, which helps them correctly understand the functions of commercial insurance and improve the problem of reverse selection. Hu et al. (19) indicated that digital finance can promote household insurance purchases by increasing residents' financial literacy and accessibility to Internet financial services.

Existing literature has explored participation in household commercial insurance. In recent years, the research perspective has expanded. However, very few authors have incorporated commercial pension insurance and medical insurance participation into a unified analysis framework. Existing studies have paid less attention to individual commercial insurance participation. There are only a few studies that conducted a macro analysis on changes in commercial insurance participation during an event. As an important guarantee for family assets and residents' health, commercial insurance has the functions of diversifying risks and organizing loss compensation and is an important part of China's insurance industry. Will demand for commercial insurance participation increase during a COVID-19 outbreak? Are changes in participation in commercial pension insurance and medical insurance consistent? What is the exact mechanism underlying this influence? This study attempts to answer these questions.

This study uses data from the China Family Panel Studies in 2014, 2016, 2018, and 2020 and the digital inclusive finance index developed by Peking University to examine the impact of the COVID-19 outbreak on individual commercial insurance and further explore its mechanism of effect. The results show that the pandemic increased the probability of participation in commercial insurance, endowment insurance, and commercial medical insurance. An analysis of the impact mechanism found that the pandemic changed risk perception, risk preference and digital finance, which increased their participation in commercial insurance.

The main contributions of this study are as follows: First, most of the existing literature analyzes the relationship between commercial insurance and COVID-19 from the perspective of the insurance industry and companies (20–22), and few have looked at the commercial insurance demands of residents. This study focuses on the changes in individuals' participation in commercial, endowment, and medical insurance after the outbreak of COVID-19. In this way, we supplement relevant literature on the impact of major public health emergencies on residents' commercial insurance participation. Second, scholars analyze the influencing factors of insurance demand from aspects such as human capital, insurance cognition, risk diversification, financial literacy, and information transparency (23–29). However, an analysis of the impact mechanisms of individual commercial insurance participation after the COVID-19 pandemic is lacking. The public's risk perception level is often used to analyze the psychological panic state (30–32). We consider risk perception and risk preference as entry points to further analyze the impact of the pandemic on commercial insurance participation, which enriches existing research in this field. Third, the COVID-19 crisis coincided with the rapid advance of various disruptive technologies, the confluence of which has been called “digital transformation” (33–35). This has accelerated the need for a digital transformation of insurance companies (35). In China, the strict quarantine made traditional offline services and processes inaccessible and digital technologies enable digital trade to substitute for face-to-face trade in the pandemic context (36, 37). This study uses the macro-digital financial development index and micro-digital financial ability to discuss the role of digital finance in the relationship between COVID-19 and commercial insurance, enriching relevant research on commercial insurance participation.

The remainder of this paper is organized as follows. Section 2 reviews related literature and presents our hypotheses. Section 3 introduces the data, variables, and models. The results are presented in Section 4. Section 5 concludes the paper.

## 2 Literature review and research hypotheses

The COVID-19 pandemic is characterized by a sudden outbreak and damage to the lungs, olfactory sensations, and pancreas, causing a high infection rate and morbidity (25, 38, 39). The public makes corresponding decisions based on risk perception to select the method of purchasing insurance (25, 40). The COVID-19 pandemic has greatly boosted demand for insurance services in the first 6 months of 2020, and this trend lasted (41). In areas more severely affected by COVID-19, prevention and control measures are stricter. The operating conditions of many enterprises have deteriorated, and unemployment has become prominent, leading to interruptions in the payment period of social insurance. The COVID-19 crisis has revealed that aspects of pandemic risk, such as business interruption and event cancelation caused by pandemic suppression, in their entirety exceed the limits of insurability (21). Forsythe et al. (42) and Caperna et al. (43) apply Google Trends to demonstrate the COVID-19-related surge in the demand for unemployment insurance in the US and EU Member States. As commercial insurance is an important supplement to social insurance, the demand for commercial insurance has increased. The basic principles of social security are “wide coverage and low level” of security. For the higher costs of isolation and medical care during the pandemic, commercial insurance, such as life insurance, accidental injury insurance, and health insurance, can compensate for individual medical costs, long-term care costs, and loss of disability income. This has become a double guarantee in the post-pandemic era. China responded to the pandemic's impact by implementing a free treatment policy and periodically reducing payments for endowment insurance, unemployment, and industrial and commercial insurance for enterprises. Additionally, it issued unemployment insurance or benefits to individuals in areas severely affected by the COVID-19 pandemic. To a certain extent, this alleviates the financial constraints and promotes participation in commercial insurance. Moreover, digital financing has enhanced social interactions among residents. The spread of collective emotions and public opinion on social media has accelerated, and the perception of risk has increased, leading to the contagion effect of panic awareness (44).

At the same time, residents pay more attention to their own health and safety and enhance their awareness of health risks, which, in turn, manifests in an increase in individual willingness to take insurance. As attention to COVID-19 and individual health anxiety has risen explosively, Internet platforms have taken advantage of this to open online inquiry platforms and pandemic-related information pages. These have played a significant role in the transparency of pandemic information, popularization of anti-pandemic knowledge, and the alleviation of anxiety. Based on the above discussion, we propose the following hypotheses:

Hypothesis 1: The outbreak of COVID-19 can promote individual commercial insurance participation.

Public health emergencies significantly improve the public's perception of risk. Threat perception after receiving and interpreting external information determines the decision-making process and protective responses (45). The degree of risk perceived by an individual is determined by his or her assessment of the threat of the COVID-19 outbreak (46, 47). According to the small probability of prospect theory, the outbreak and spread of COVID-19 will strengthen individuals' risk perceptions and incline them to purchase insurance. Under the impact of the pandemic, risk perception is more sensitive, and there is hope for greater utility from insurance. Moreover, according to imprinting theory, major external events experienced by individuals are projected onto future behavioral choices (48). For instance, Botzen and van den Bergh (49) confirmed that the stronger individuals perceive their flood risk, the greater the demand for flood insurance. Peng et al. (50) found that disaster shocks directly impact farmers' willingness to buy insurance and indirectly impact their willingness through risk perception. As a public health emergency, COVID-19 may continue to affect risk perceptions and change demand for insurance.

Risk aversion is a strong predictor of individuals' protective behavior (51). Assuming rational and complete markets, investors choose to allocate diversified assets to avoid risks. The degree of diversification in risk asset allocation is related to investors' risk preferences. Prospect theory posits that, in a loss situation, people generally prefer risk. The outlook theory proposes that when expectations are uncertain, people tend to prefer risks. Therefore, in the context of COVID-19, individuals are extremely prone to preferring risk. As commercial insurance has both protection and investment attributes, it may be favored by risk-prone individuals (52–54). Giné et al. (52) explained that risk-averse households are less likely to purchase rainfall insurance. Cardak and Wilkins (53) discovered that household superannuation and risky financial assets are complementary rather than substitutes. Sun and Xiong (55) found that households with an appetite for risk are more likely to purchase commercial insurance. Xie et al. (56) argued that commercial insurance, as a type of insurance with both investment and protection functions and no compulsory purchase and may be more popular among risk-preferring people. Risk-seeker individuals are more likely to have access to financial investment market information, such as stocks, and have a stronger perception of risk. Familial groups have a higher subjective risk preference and a higher demand for and awareness of insurance.

At present, the main prevention and control measures for COVID-19 are isolation, blockade, and social distancing. These provide development opportunities for digital finance (17, 57, 58). Unlike traditional finance, digital finance can guide the effective allocation of financial resources and help improve enterprises' risk resistance and survival ability through credit. Digital finance not only promotes the balanced development of the regional economy, innovation, and entrepreneurship but also enhances residents' income and boosts consumption growth (59, 60). The COVID-19 crisis coincides with the rapid advancement of various disruptive technologies (33–35), which has accelerated the need for a digital transformation of insurance companies (35). In China, the strict quarantine led to the inaccessibility of traditional offline services and processes. Digital technologies allow digital trade to substitute face-to-face trade in the pandemic context (36, 37). The development of digital finance forced the transformation and upgrading of the traditional insurance industry during COVID-19, effectively broadening the service boundaries of relevant financial institutions. It reduced the threshold for enjoying financial services and also reduced the cost of information and operations to participate in commercial insurance (18), which also improved the availability of commercial insurance (61). Insurance companies rely on big data, cloud computing, and other technologies to sell insurance products in the cloud, which not only controls operating costs but also accurately conducts credit ratings, risk pricing, and default forecasts based on consumer transaction records to improve the matching of supply and demand (62). Simultaneously, digital finance has promoted participation in commercial insurance by improving the availability of commercial insurance, reducing transaction costs, and improving financial literacy. Previous research confirmed that residents face higher information search costs when participating in financial markets. The Internet makes it easier for residents to obtain financial knowledge and improve their financial literacy (63–65), thereby improving residents' awareness of insurance and helping increase commercial insurance participation (66). Additionally, those with a higher level of financial literacy are more likely to think about their financial needs after retirement, have long-term financial plans, and hold private pensions (67). Based on the above discussion, we propose the following hypothesis:

Hypothesis 2: The outbreak of COVID-19 improves residents' risk perception, risk preference and digital finance, thereby promoting participation in commercial insurance.

## 3 Methods, data, and descriptive statistics

### 3.1 Empirical model

COVID-19 can be viewed as a quasi-natural experiment. Its impact on residents' commercial insurance participation is affected by multiple factors, such as individuals, families, and economic and social conditions, resulting in biased results. To reduce the interference of other factors, a difference-in-differences model was employed. Referring to Athey and Imbens (68), the model was set as follows:


(1) $$\begin{array}{l} Logit(insurance_{it}=1)=\alpha_{0}+\alpha_{1}treat_{i}\times time_{t}+\\ \;\alpha_{2}control_{it}+ind_{i}+city_{it}+year_{t}+\varepsilon_{it} \end{array}$$

In Equation (1), *insurance*_*it*_ is the commercial insurance participation variable of the individual *i* in year *t*, including general, endowment and medical commercial insurance. The group dummy variable and time dummy variable are represented by *treat*_*i*_ and *time*_*t*_, respectively; vector *control*_*it*_ is a set of control variables; *ind*_*i*_, *city*_*it*_, and *year*_*t*_ denote the individual fixed effects of the individual *i* that do not vary over time, area fixed effects of the individual *i* in year *t* over time and individual and year-fixed effects of the year *t* that do not vary among individuals, respectively; ε_*it*_ denotes the random disturbance term. This study focused on the coefficient α_1_, which reflects the net impact of the impact of the COVID-19 outbreak on commercial insurance participation.

In addition to the direct effect captured by Equation (1), this research investigates the mechanism of COVID-19′s impact on residents' commercial insurance participation. This study constructs the following model:


(2) $$\begin{array}{l} mechanism_{it}=\beta_{0}+\beta_{1}treat_{i}\times time_{t}+\beta_{2}control_{it}+\\ \;ind_{i}+city_{it}+year_{t}+\varepsilon_{it} \end{array}$$

(3) $$\begin{array}{l} \;Logit(insurance_{it}=1)=\gamma_{0}+\gamma_{1}treat_{i}\times time_{t}+\\ \gamma_{2}mechanism_{it}+\gamma_{3}control_{it}+ind_{i}+city_{it}+year_{t}+\varepsilon_{it} \end{array}$$

In Equation (2), *mechanism*_*it*_ is the influence mechanism of the individual *i* in year *t*, including risk perception, risk preference and digital finance. The significance of the regression coefficients of α_1_, β_1_, γ_1_, and γ_2_ in Equations (1–3) are used to determine the existence of mediating effects.

### 3.2 Definition of variables

#### 3.2.1 Insurance participation variable

The commercial insurance participation variables include: general (*dem*), endowment (*endowment*) and medical (*medical*). The value of *dem* is 1 if the individual buys commercial endowment insurance or commercial medical insurance and 0 otherwise. The value of *endowment* is 1 if the individual buys commercial endowment insurance and 0 otherwise. The value of *medical* is 1 if the individual buys commercial medical insurance; otherwise, it is 0.

#### 3.2.2 Group dummy variable and time dummy variable

Based on the number of confirmed COVID-19 cases, the severity of impact in the sample area was identified to determine the group dummy variable *treat*. According to the sample interview time, the provinces with more than 1,000 confirmed cases by August 31, 2020, were listed as the regions severely affected by COVID-19, namely, the experimental group. Under this standard, the samples of individuals in Hubei, Guangdong, Henan, Zhejiang, and Hunan provinces were classified into experimental groups^1^ (*treat* = 1); individuals in the other provinces were in the control groups (*treat* = 0).

The time dummy variable *time* is determined by the time of the COVID-19 outbreak. If the sample is from 2014, 2016, and 2018, the value of *time* is 0; otherwise, it is 1.

#### 3.2.3 Mechanism variable

Referring to Jia et al. (69), this study used factor analysis to measure the *risk perception* from the perspective of emotions and cognition regarding various risks in various fields, including environmental protection, the wealth gap, employment, education, medical care, housing, social security, and government integrity. The results showed that the KMO value was 0.9098, which was suitable for factor analysis.

Referring to Anderson and Mellor (70), this study used individual's drinking (whether more than three times a week), exercise (length of each exercise), and smoking (cigarettes per day) to measure *risk preference*. We standardized the answers to these three questions and used the mean to generate the risk preference variable.

Referring to Qin et al. (71), Hu et al. (72), Yi et al. (73), Zhu et al. (74), and Huang et al. (75), this study used digital financial data at the regional level and matched it at an individual level. The study utilized the digital insurance use depth index at both prefecture and city levels as proxy variables to gauge the level of digital finance development. At the same time, to reduce endogeneity problems caused by reverse causality, we adopted a one-period lagged digital finance index.

In addition, we selected five questions from the questionnaire concerning whether or not Internet browsing, Internet-based learning, online socializing, online entertainment, and online shopping were conducted. We used an iterative main factor method to measure individual digital finance useability for the sample's answers to these questions. The results showed that the KMO value was 0.8649, which was suitable for factor analysis.

#### 3.2.4 Control variables

Based on the existing literature and field experience, we included three levels of control variables (3, 19, 76–79). First, we included individual characteristics, such as sex, age, marriage status, education, health status and household or “hukou” status (urban or rural). Second, at the household level, we included economic and demographic characteristics. This included fml (total household population), income (the logarithmic of the average annual household income per member), and the proportion of unhealthy household members. We also included the Old Dependency Ratio (ODR), defined as the proportion of those aged 65 and older in relation to the working-age population, and Child Dependency Ratio (CDR), defined as the proportion of children under 14 years old in relation to the working-age population. Third, at the macro level, we considered area-fixed effects. Lastly, we considered individual fixed effects and year-fixed effects. The specific details of the variables are presented in Table 1.

**Table 1:** Variable definition.

**Variable**	**Definition**
Dem	If the individual participates in commercial pension insurance or commercial medical insurance, the dem is 1; otherwise, the dem is 0
Endowment	If the individual participates in commercial pension insurance, the endowment is 1; otherwise, the endowment is 0
Medical	If the individual participates in commercial medical insurance, the medical is 1; otherwise, the medical is 0
Treat	Experimental group 1, control group 0
Time	Year = 2020, time 1; otherwise, 0
Risk perception	The average of standardized risk perception values in environmental protection, the gap between rich and poor, employment, education, medical care, housing, social security, and government integrity
Risk preference	The average of standardized values in whether drink more than 3 times a week, the length of each exercise, and the amount of smoking per day
Insurance	One-period lagged the digital insurance use depth index
Df	Generated by the factor analysis method
Sex	Male 1, female 0
Age	Year of investigation-Year of birth
Age2	Square of age/100
Marriage	Unmarried 1, married 2, cohabiting 3, divorced 4, widowed 5
Edu	The highest individual education is illiterate/semi-illiterate 0, primary school 1, junior high school 2, high school/secondary school/technical school/vocational high school 3, college 4, bachelor degree and above 5
Health	Excellent 1, very good 2, good 3, fair 4, poor 5
Hukou	Town 1, rural 0
Fml	Total household population
Income	The logarithm of the ratio between the total household income and total household population
Unhealthy	The proportion of unhealthy members in the total household population
Odr	The proportion of the number of people over 65 years old in the family in the total household population
Cdr	The proportion of number of people under 14 years old in the family in the total household population

### 3.3 Data sources

This study used data from the China Family Panel Studies in 2014, 2016, 2018, and 2020 and the Peking University Digital Financial Inclusion Index Database. After using the Peking University Digital Financial Inclusion Index Database to match the districts and counties of the China Family Panel Studies, we removed the 2020 samples interviewed in September and after September and the samples with missing data and obtained 61,351 samples. Simultaneously, we winsorized continuous annual variables by 1 percentile each year to mitigate the influence of outliers.

### 3.4 Summary statistics

Table 2 presents the descriptive statistics of the variables. Currently, the participation rate of commercial insurance in China is relatively low. The average participation rates for general, endowment, and medical commercial insurance are 0.017, 0.007, and 0.010, respectively. The participation rate of individuals in commercial endowment insurance and commercial medical insurance are both low, reflecting the current insufficient participation of residents in commercial insurance, which is in line with the reality in China. The results of the *t*-test showed that dem, endowment and medical variables were significant, indicating notable groups before and after the COVID-19 outbreak. The general, endowment and medical commercial insurance participation rates of the group after COVID-19 were greater than those before the outbreak. The remaining variables in the sample were similar to the descriptive statistical results in the existing literature.

**Table 2:** Summary statistics.

**Variable**	**Mean**	**MIN**	**P25**	**P50**	**P75**	**MAX**	**SD**	**Before COVID-19**	**After COVID-19**	***T*-test**
Dem	0.017	0	0	0	0	1	0.130	0.016	0.0220	−0.005^***^
Endowment	0.007	0	0	0	0	1	0.086	0.007	0.009	−0.002^**^
Medical	0.010	0	0	0	0	1	0.102	0.010	0.013	−0.003^***^
Risk perception	0.66	0.125	0.525	0.663	0.813	1	0.196	0.669	0.618	0.050^***^
Risk preference	0.081	0	0	0.001	0.067	0.433	0.140	0.0810	0.0800	0.002
Insurance	79.26	8.82	60.22	75.9	96.32	170.5	29.98	89.95	114.0	−24.052^***^
Df	−0.035	−0.871	−0.871	−0.792	1.02	1.412	0.955	77.69	86.84	−9.152^***^
Sex	0.558	0	0	1	1	1	0.497	−0.113	0.363	−0.476^***^
Age	48.2	18	36	49	60	81	15.64	0.571	0.493	0.078^***^
Age2	25.68	3.24	12.96	24.01	36	65.61	15.26	47.84	50.05	−2.202^***^
Marriage	2.102	1	2	2	2	5	0.800	25.36	27.35	−1.990^***^
Edu	1.778	0	1	2	3	5	1.393	2.097	2.127	−0.030^***^
Health	3.046	1	2	3	4	5	1.213	1.756	1.892	−0.136^***^
Hukou	0.298	0	0	0	1	1	0.457	3.052	3.015	0.037^***^
Fml	4.263	1	3	4	5	11	1.919	0.298	0.299	−0.001
Income	9.511	6.652	8.923	9.567	10.14	11.92	0.975	4.266	4.244	0.0230
Unhealthy	0.13	0	0	0	0.2	1	0.212	9.463	9.758	−0.296^***^
Odr	0.153	0	0	0	0.25	1	0.268	0.130	0.131	−0.001
Cdr	0.132	0	0	0	0.25	0.5	0.158	0.148	0.178	−0.030^***^

The symbols ^*^, ^**^, and ^***^ denote statistical significance at the 10, 5, and 1% levels, respectively.

## 4 Results

### 4.1 Analysis of baseline regression results

Columns (1)–(3) of Table 3 report the regression results of Model (1). The results showed that in areas that were affected more by COVID-19, the likelihood of individuals participating in commercial insurance, commercial endowment insurance, and commercial medical insurance increased significantly after the COVID-19 outbreak. When the values of the other variables remained unchanged, the probability of individuals in the experimental group purchasing commercial insurance was 0.79% higher than that of the control group, and the probabilities of purchasing commercial endowment insurance and commercial medical insurance were 0.42 and 0.41%, respectively. Therefore, Hypothesis 1 was supported. The comprehensive empirical results showed that COVID-19 had a greater positive impact on participation in general, endowment, and medical commercial insurance in the experimental group. Although COVID-19 has caused inconvenience to the production and lives of people across the country, the sample of provinces with a large number of confirmed cases is more sensitive to risk perception. The state has subsidized social security to ease financial constraints, increasing the demand for commercial insurance participation.

**Table 3:** Baseline results.

	**Dem**	**Endowment**	**Medical**
	**(1)**	**(2)**	**(3)**
Treat × time	0.0079^***^	0.0042^***^	0.0041^**^
	(3.2228)	(2.6044)	(2.1148)
Sex	0.0027^**^	0.0015^**^	0.0016^*^
	(2.4141)	(1.9993)	(1.8689)
Age	0.0010^***^	0.0019^***^	0.0000
	(3.8122)	(7.2557)	(0.2663)
Age2	−0.0018^***^	−0.0027^***^	−0.0004^*^
	(−5.9545)	(−8.1333)	(−1.9268)
Marriage	0.0016^*^	−0.0000	0.0012^*^
	(1.9015)	(−0.0000)	(1.9222)
Edu	0.0020^***^	0.0004	0.0017^***^
	(4.2304)	(1.4043)	(4.6933)
Health	0.0027^***^	0.0014^***^	0.0014^***^
	(4.8078)	(3.6378)	(3.2698)
Hukou	0.0115^***^	0.0045^***^	0.0079^***^
	(8.9829)	(5.3209)	(7.6511)
Fml	−0.0008^**^	0.0001	−0.0008^***^
	(−2.1738)	(0.6364)	(−2.6625)
Income	0.0054^***^	0.0023^***^	0.0036^***^
	(7.5097)	(4.8122)	(6.2370)
Unhealthy	−0.0175^***^	−0.0113^***^	−0.0071^***^
	(−4.7459)	(−3.9423)	(−2.6911)
Odr	0.0029	−0.0106^***^	0.0033
	(0.8816)	(−3.0781)	(1.4347)
Cdr	−0.0022	−0.0046^*^	−0.0014
	(−0.5822)	(−1.8435)	(−0.4666)
Individual	Y	Y	Y
Area	Y	Y	Y
Year	Y	Y	Y
*N*	61,351	61,351	61,351
*R*2	0.0823	0.0997	0.0785

^*^, ^**^, and ^***^ denote statistical significance at the 10, 5, and 1% levels, respectively. The values in parentheses are robust standard errors.

### 4.2 Test of parallel trend assumption

**Figure 1:**
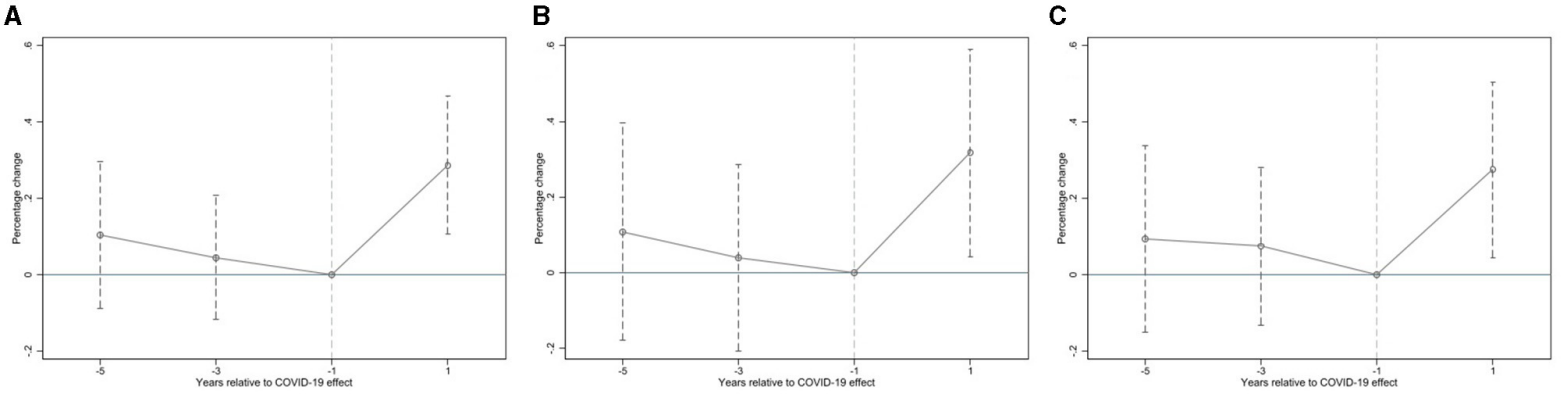
Test the parallel trend hypotheses. **(A)** represents parallel trend test for dem, **(B)** showcases parallel trend test for endowment, and **(C)** delineates parallel trend test for medical.

The parallel pre-trend assumption is the key to the validity of the DID approach. We ran falsification tests using the dynamic model to validate the pretreatment parallel-trend assumption. Specifically, we regarded 2019 as the base period of COVID-19, before and after representing the period before and after COVID-19, respectively; therefore, pre3 represents the 3 years before COVID-19. To avoid multiple collinearity problems, we deleted pre1. Figure 1 and Table 4 test the parallel trend hypotheses. The results show that the interaction term coefficients were not significant before the outbreak of COVID-19, but they were significantly positive after the outbreak, indicating that there was no significant difference between the experimental and control groups before COVID-19. This suggests that the parallel trend assumption for the difference-in-differences approach is plausible.

**Table 4:** Parallel trends test.

	**Dem**	**Endowment**	**Medical**
	**(1)**	**(2)**	**(3)**
Pre5 × treat	0.0017	0.0008	0.0010
	(1.0716)	(0.7407)	(0.7470)
Pre3 × treat	0.0007	0.0003	0.0008
	(0.5475)	(0.3168)	(0.7070)
After1 × treat	0.0047^***^	0.0023^**^	0.0028^**^
	(3.0902)	(2.2520)	(2.3373)
Control variables	Y	Y	Y
Individual	Y	Y	Y
Area	Y	Y	Y
Year	Y	Y	Y
*N*	61,351	61,351	61,351
*R*2	0.0814	0.0985	0.0779

^*^, ^**^, and ^***^ denote statistical significance at the 10, 5, and 1% levels, respectively. The values in parentheses are robust standard errors.

### 4.3 Robustness tests

#### 4.3.1 Suppose the COVID-19 outbreak in 2016 and 2018

Referring to Huang (80), this study assumed that the COVID-19 outbreak in 2016 and 2018 generated the virtual variable time 2016 and 2018 to test the impact of COVID-19 on commercial insurance participation. The regression results in Table 5 show that the assumed impact of the pandemic in other years does not significantly affect commercial insurance participation, indicating that the baseline regression results are relatively less disturbed by inherent differences and unobservable variables between the experimental and control groups.

**Table 5:** Placebo test.

	**Dem**	**Endowment**	**Medical**	**Dem**	**Endowment**	**Medical**
	**(1)**	**(2)**	**(3)**	**(4)**	**(5)**	**(6)**
Treat × time2016	0.0011	0.0013	−0.0002			
	(0.7312)	(1.3527)	(−0.1857)			
Treat × time2018				0.0009	0.00002	0.0008
				(0.6393)	(0.0164)	(0.7044)
Control variables	Y	Y	Y	Y	Y	Y
Individual	Y	Y	Y	Y	Y	Y
Area	Y	Y	Y	Y	Y	Y
Year	Y	Y	Y	Y	Y	Y
*N*	61,351	61,351	61,351	61,351	61,351	61,351
*R*2	0.0814	0.0988	0.0779	0.0814	0.0985	0.0780

^*^, ^**^, and ^***^ denote statistical significance at the 10, 5, and 1% levels, respectively. The values in parentheses are robust standard errors.

#### 4.3.2 Randomly selected experimental group

**Figure 2:**
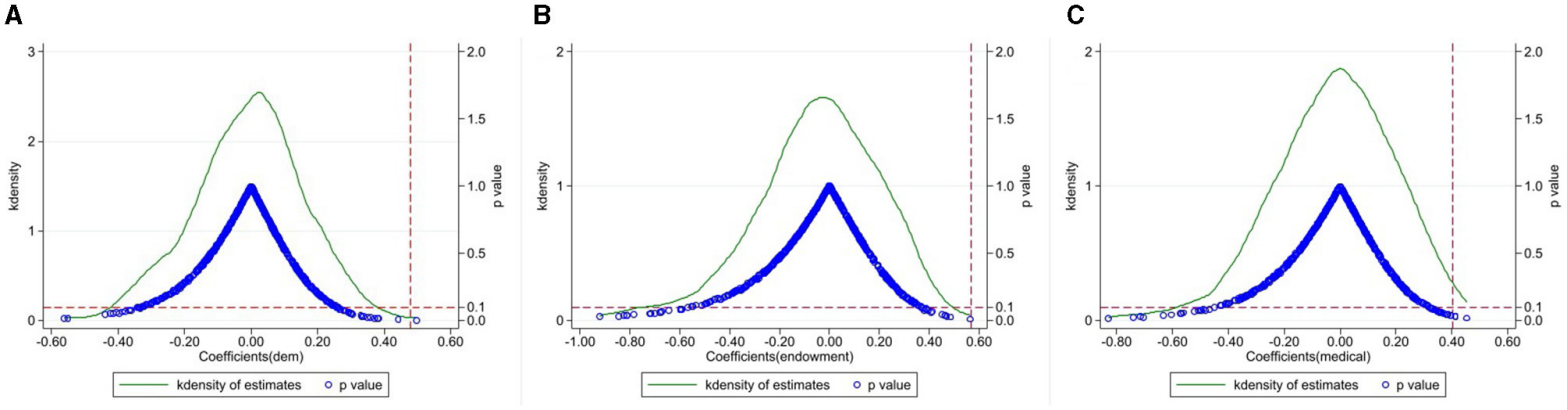
This figure shows the placebo test by randomly generating treatment groups. **(A)** represents placebo test in dem, **(B)** showcases placebo test in endowment, and **(C)** delineates placebo test in medical.

To alleviate the influence of other accidental factors on the empirical results, we followed Li et al. (81) and conducted a placebo test by randomly generating treatment groups. Specifically, by randomly selecting individuals in the treatment group, a pseudo-policy dummy variable (Treat^*^) was created to replace Treat in Model (1) for the baseline regression. This study repeated the above random selection 500 times and obtained the kernel density figure of the coefficients and the *p*-value of Treat^*^×Post. As shown in Figure 2, most *p*-values are >0.1, and the actual estimates are significant outliers in the placebo test. Therefore, random factors are unlikely to drive the results of this study, and the placebo test supported the results of this study.

#### 4.3.3 Potential confounding policies

If other concurrent policies affect insurance participation, the estimates may be biased. Based on a thorough review of possibly related policies, we believe that the most likely candidates are the “Healthy China 2030” planning outlines and the social security reduction and exemption policy. The planning outlines encourage the development of commercial health insurance, the implementation of tax incentives, and the active participation of enterprises and individuals. In response to the impact of COVID-19, China issued a social security reduction and exemption policy for enterprises of different sizes and regions in February 2020, including relief of pension, unemployment and employment injury insurance. We set policy dummy variables based on the sample interview time and incorporated them into the baseline regression. As shown in Table 6, the impact coefficient of the COVID-19 outbreak on *dem, endowment*, and *medical* are all significant, indicating the robustness of the conclusions of this study.

**Table 6:** Robustness check.

	**Dem**	**Endowment**	**Medical**
	**(1)**	**(2)**	**(3)**
Treat × time	0.0079^***^	0.0042^***^	0.0041^**^
	(3.2228)	(2.6045)	(2.1146)
Policy 1	0.0082	−0.0010	0.0071^**^
	(1.6422)	(−0.2392)	(1.9832)
Policy 2	−0.0075	0.0013	−0.0064^*^
	(−1.4064)	(0.2800)	(−1.6525)
Control variables	Y	Y	Y
Individual	Y	Y	Y
Area	Y	Y	Y
Year	Y	Y	Y
*N*	61,351	61,351	61,351
*R*2	0.0825	0.0997	0.0790

^*^, ^**^, and ^***^ denote statistical significance at the 10, 5, and 1% levels, respectively. The values in parentheses are robust standard errors.

#### 4.3.4 Endogeneity

The possible estimation bias due to measurement errors needs to be treated with caution. In addition, the baseline model may have contained missing variables. Therefore, we sought suitable instrumental variables to address this concern. Drawing on Buchak et al. (82), we selected the linear distance of the sample district from Wuhan, thereby using physical location as a tool variable. In the early days of COVID-19, Wuhan was the city most seriously affected in China. If a city is located closer to Wuhan, it is more likely to be affected by COVID-19. At the same time, it is difficult for the straight-line distance of districts and counties from Wuhan to directly affect an individual's commercial insurance participation; therefore, the instrumental variable meets the exogenous requirements.

The instrumental variable estimates are listed in Table 7. The K-P LM statistic was 15,575.28, and the *p*-value was 0.000, which means that there was no under-identification problem for the instrumental variables. The C-D Wald *F-*statistic is greater than the critical value of 16.38 at the 10% level calculated by Stock and Yogo (83), indicating that there is no problem with weak instrumental variables. Column (1) shows that the regression coefficient of the first stage is significant, proving that the instrumental variable is related to the group variable. The results of the second-stage (2)–(4) columns show that the COVID-19 outbreak significantly promoted commercial insurance participation. Therefore, after eliminating the interference of endogenous problems in the model, the basic conclusions of previous studies are robust and reliable.

**Table 7:** Instrumental variable regression.

	**First stage**	**Second stage**		
	**Treat**	**Dem**	**Endowment**	**Medical**
	**(1)**	**(2)**	**(3)**	**(4)**
IV	−0.0009^***^			
	(−2.1e+02)			
Treat × time		0.0010^***^	0.0007^**^	0.0005^*^
		(2.8941)	(2.4995)	(1.8300)
Kleibergen-Paap rk LM statistic	15,575.28^***^	—	—	—
Cragg-Donald Wald F statistic	20,172.11	—	—	—
Control variables	Y	Y	Y	Y
Individual	Y	Y	Y	Y
Area	Y	Y	Y	Y
Year	Y	Y	Y	Y
*N*	61,351	61,351	61,351	61,351
*R*2	0.4723	0.0824	0.1014	0.0784

^*^, ^**^, and ^***^ denote statistical significance at the 10, 5, and 1% levels, respectively. The values in parentheses are robust standard errors.

### 4.4 Mechanism analysis

In the above steps, we showed that COVID-19 promoted participation in commercial insurance. Subsequently, we used Models (2, 3) to test the mechanism of COVID-19's impact on commercial insurance participation. The regression results are shown in Table 8. After including the mediating variable, the COVID-19 outbreak promoted individual participation in commercial insurance by influencing risk perception, risk preference, and digital finance. The above three mechanisms were tested with the mediating effect model, and the regression results show that the mediating effect holds. This indicates that the COVID-19 outbreak promotes an individual's participation in commercial insurance through the above three paths.

**Table 8:** Mechanism test.

	**Risk perception**	**Dem**	**Endowment**	**Medical**	**Risk preference**	**Dem**	**Endowment**	**Medical**	**Insurance**	**Dem**	**Endowment**	**Medical**	**Df**	**Dem**	**Endowment**	**Medical**
	**(1)**	**(2)**	**(3)**	**(4)**	**(5)**	**(6)**	**(7)**	**(8)**	**(9)**	**(10)**	**(11)**	**(12)**	**(13)**	**(14)**	**(15)**	**(16)**
Treat × time	0.0820^***^	0.0078^***^	0.0041^**^	0.0040^**^	0.0051^*^	0.0078^***^	0.0041^***^	0.0041^**^	1.9479^***^	0.0065^***^	0.0033^**^	0.0033^**^	0.0318^*^	0.0076^***^	0.0041^**^	0.0039^**^
	(3.9338)	(3.183)	(2.576)	(2.080)	(1.9223)	(3.201)	(2.587)	(2.098)	(11.602)	(2.759)	(2.188)	(2.188)	(1.785)	(3.111)	(2.540)	(2.032)
Risk perception		0.0023^***^	0.0011^**^	0.0014^**^												
		(3.221)	(2.202)	(2.489)												
Risk preference						0.0091^**^	0.0042^*^	0.0061^**^								
						(2.389)	(1.674)	(2.033)								
Insurance										0.0001^***^	0.00004^**^	0.00004^**^				
										(3.166)	(2.291)	(2.291)				
Df														0.0039^***^	0.0011^**^	0.0027^***^
														(5.251)	(2.386)	(4.595)
Control variables	Y	Y	Y	Y	Y	Y	Y	Y	Y	Y	Y	Y	Y	Y	Y	Y
Individual	Y	Y	Y	Y	Y	Y	Y	Y	Y	Y	Y	Y	Y	Y	Y	Y
Area	Y	Y	Y	Y	Y	Y	Y	Y	Y	Y	Y	Y	Y	Y	Y	Y
Year	Y	Y	Y	Y	Y	Y	Y	Y	Y	Y	Y	Y	Y	Y	Y	Y
*N*	61,350	61,351	61,351	61,351	61,350	61,351	61,351	61,351	55,858	55,858	55,858	55,858	61,350	61,350	61,350	61,350
*R*2	0.129	0.0833	0.1006	0.0794	0.127	0.0828	0.1002	0.0791	0.912	0.0813	0.1008	0.1008	0.432	0.0850	0.1008	0.0816

^*^, ^**^, and ^***^ denote statistical significance at the 10, 5, and 1% levels, respectively. The values in parentheses are robust standard errors.

Column (1) shows that the coefficient of treatment × time is significantly positive at the 1% level, indicating that COVID-19 has a positive stimulatory effect on risk perception. Columns (2)–(4) show that the coefficient of treatment × time and risk perception are significantly positive. The regression results show that the mediating effect holds. During the initial phase of the COVID-19 outbreak, the number of infections and deaths continued to increase, triggering a certain degree of risk awareness among the people. Moreover, the rapid dissemination of information related to COVID-19 in the digital era further magnified the public's risk perception through psychological, social, and cultural interactions. This risk perception was transformed into practical insurance needs. The regression results show that the mediating effect holds.

Column (5) shows that the COVID-19 outbreak has a significantly positive impact on risk preference. Columns (6)–(8) show that the coefficient of treatment × time and risk preference are significantly positive. The regression results show that the mediating effect holds. The impact of COVID-19 made residents gradually aware of the risk of loss of income caused by major diseases to individuals and families, causing public panic and anxiety and a significant increase in risk preference for income, thereby stimulating commercial insurance participation.

Columns (9) and (13) show that the coefficients of treatment × time are both significantly positive, indicating that the COVID-19 outbreak has a significantly positive impact on digital finance. Columns (10)–(12), (14)–(16) show that the coefficient of treatment × time and digital finance are all significantly positive. The regression results show that the mediating effect holds. The COVID-19 outbreak has promoted the rapid development of digital finance, broadened commercial insurance purchase channels and enhanced individuals' financial knowledge, social interaction, and financial trust. Simultaneously, the development of digital finance prompted insurance institutions to continuously develop a network of commercial insurance products, resulting in lower search and transaction costs for residents, thereby promoting their participation in commercial insurance. In addition, pandemic prevention and control measures have led to an increase in online exchanges, and the herding effect of commercial insurance participation has become more significant. Thus, individuals have increased their enthusiasm to participate in commercial insurance purchases.

Thus, Hypothesis 2 is confirmed.

### 4.5 Analysis of heterogeneity

Considering the heterogeneity of commercial insurance participation in several dimensions, we explored the diverse effects of possible heterogeneity on commercial insurance participation.

#### 4.5.1 Urban and rural individuals

COVID-19 prevention and control measures restrict the movement of people to a certain extent and have a significant impact on rural areas. Therefore, insurance demand may differ between urban and rural areas. This study examined the heterogeneous effects of the COVID-19 outbreak on commercial insurance participation among people with different household registrations.

Table 9 presents the estimated results. The results show that the COVID-19 outbreak had a significant positive impact on the commercial insurance and commercial medical insurance participation of urban individuals. Compared with rural individuals, urban individuals have higher incomes and better social security systems; therefore, they are more inclined to purchase commercial insurance to obtain risk resistance, especially when the demand for commercial medical insurance has increased. Columns (4)–(6) show that the impact coefficient of the COVID-19 outbreak on commercial endowment insurance in rural areas is not significant but is still positive. This may be because COVID-19 has resulted in the vulnerability of the rural economic system being even more significant. Additionally, the basic coverage offered by rural endowment and medical insurance provides limited protection, prompting an increased demand for commercial endowment and medical insurance.

**Table 9:** Heterogeneity test of urban and rural individuals.

	**Urban individuals**			**Rural individuals**		
	**Dem**	**Endowment**	**Medical**	**Dem**	**Endowment**	**Medical**
	**(1)**	**(2)**	**(3)**	**(4)**	**(5)**	**(6)**
Treat × time	0.0183^***^	0.0083^**^	0.0097^**^	0.0027	0.0019	0.0017
	(3.1128)	(2.2522)	(1.9678)	(1.0517)	(1.0862)	(0.9218)
Control variables	Y	Y	Y	Y	Y	Y
Individual	Y	Y	Y	Y	Y	Y
Area	Y	Y	Y	Y	Y	Y
Year	Y	Y	Y	Y	Y	Y
N	18,270	18,270	18,270	43,081	43,081	43,081
R2	0.0578	0.1079	0.0466	0.0557	0.0703	0.0443

^*^, ^**^, and ^***^ denote statistical significance at the 10%, 5%, and 1% levels, respectively. The values in parentheses are robust standard errors.

#### 4.5.2 Regional commercial insurance participation rate

As a type of financial decision-making, commercial insurance decision-making may have a “neighborhood effect,” affecting people in the same community. Districts and counties with different commercial insurance participation and different rates of COVID-19 differ from each other. In locations with high insurance participation rates, the positive impact of COVID-19 on commercial insurance participation may be magnified due to the “neighborhood effects.” We regarded districts and counties. We categorized districts and counties with commercial insurance participation rates higher than the province's average as having high participation rates, while those below the average were considered to have low participation rates.

Table 10 shows that in locations with high commercial insurance participation rates, the effect coefficients of the COVID-19 outbreak on commercial insurance participation and commercial endowment insurance participation were 0.0136 and 0.0076, respectively. This is positive and significant, indicating that during the COVID-19 outbreak, there was a “neighborhood effect” on commercial insurance decision-making.

**Table 10:** Heterogeneity test of different regional commercial insurance participation rates.

	**Districts and counties with high insurance participation rates**			**Districts and counties with low insurance participation rates**		
	**Dem**	**Endowment**	**Medical**	**Dem**	**Endowment**	**Medical**
	**(1)**	**(2)**	**(3)**	**(4)**	**(5)**	**(6)**
Treat × time	0.0136^***^	0.0076^**^	0.0056	0.0026	0.0009	0.0024
	(2.6639)	(2.3337)	(1.3633)	(1.2843)	(0.5750)	(1.5918)
Control variables	Y	Y	Y	Y	Y	Y
Individual	Y	Y	Y	Y	Y	Y
Area	Y	Y	Y	Y	Y	Y
Year	Y	Y	Y	Y	Y	Y
*N*	26,079	26,079	26,079	35,272	35,272	35,272
*R*2	0.0745	0.1052	0.0632	0.0706	0.0699	0.0908

^*^, ^**^, and ^***^ denote statistical significance at the 10, 5, and 1% levels, respectively. The values in parentheses are robust standard errors.

#### 4.5.3 Sex

Previous research has suggested that women perceive risks associated with the COVID-19 pandemic more than males and, thus, were more active in practicing prevention in response to the pandemic (84). Women respond more to COVID-19 compared to men and become more risk-averse in the specific Social and Experience Seeking domains (85). Besides, the convenience of digital finance makes women get access to financial services more easily (81). Therefore, there may be differences in demand for commercial insurance between females and males. This study examined the heterogeneous effects of the COVID-19 outbreak on commercial insurance.

Table 11 shows that for females, the effect coefficients of the COVID-19 outbreak on general, endowment and medical commercial insurance participation were both positive and significant. This indicates that during the COVID-19 outbreak, they were more willing to buy commercial insurance.

**Table 11:** Heterogeneity test of different sex.

	**Female**			**Male**		
	**Dem**	**Endowment**	**Medical**	**Dem**	**Endowment**	**Medical**
	**(1)**	**(2)**	**(3)**	**(4)**	**(5)**	**(6)**
Treat × time	0.0111^***^	0.0047^**^	0.0074^***^	0.0043	0.0038	−0.0003
	(3.6737)	(2.3077)	(3.1275)	(1.0841)	(1.5605)	(−0.0788)
Other control variables	Y	Y	Y	Y	Y	Y
Individual	Y	Y	Y	Y	Y	Y
Area	Y	Y	Y	Y	Y	Y
Year	Y	Y	Y	Y	Y	Y
*N*	27,108	27,108	27,108	34,243	34,243	34,243
*R*2	0.0843	0.0924	0.0849	0.0712	0.0985	0.0642

^*^, ^**^, and ^***^ denote statistical significance at the 10, 5, and 1% levels, respectively. The values in parentheses are robust standard errors.

#### 4.5.4 Per capita household income level

Generally, low-income populations are more vulnerable to disaster situations, while higher-income families are better prepared than lower-income families (86). Low-income residents may have a limited understanding of insurance and risk management, and this may affect their commercial insurance participation. Therefore, insurance demand may differ between low-income and higher-income populations. This study examined the heterogeneous effects of the COVID-19 outbreak on commercial insurance. We categorized individuals with per capita household income levels higher than the average in districts and counties where the sample is located as a high-income group, while those below the average were considered as a low-income group.

Table 12 shows that for the group with high per capita household income level, the effect coefficients of the COVID-19 outbreak on general, endowment and medical commercial insurance participation were both positive and significant. However, the low-income populations are all not significant, indicating increased policy allowance and efforts are needed to improve participation in commercial insurance in low-income populations.

**Table 12:** Heterogeneity test of different per capita household income level.

	**High-income group**			**Low-income group**		
	**Dem**	**Endowment**	**Medical**	**Dem**	**Endowment**	**Medical**
	**(1)**	**(2)**	**(3)**	**(4)**	**(5)**	**(6)**
Treat × time	0.0124^***^	0.0071^***^	0.0058^**^	0.0007	−0.0024	0.0028
	(3.5319)	(3.1411)	(2.0543)	(0.1396)	(−0.6018)	(0.6908)
Other control variables	Y	Y	Y	Y	Y	Y
Individual	Y	Y	Y	Y	Y	Y
Area	Y	Y	Y	Y	Y	Y
Year	Y	Y	Y	Y	Y	Y
*N*	22,534	22,534	22,534	38,816	38,816	38,816
*R*2	0.0625	0.0930	0.0558	0.0793	0.0978	0.0754

^*^, ^**^, and ^***^ denote statistical significance at the 10, 5, and 1% levels, respectively. The values in parentheses are robust standard errors.

## 5 Conclusion

Commercial insurance employs market mechanisms for risk management, playing an important role in market-oriented risk transfer mechanisms. Participating in commercial insurance can help individuals mitigate future uncertainties, smooth consumption and welfare, and alleviate economic vulnerability. This study used a sample from China Family Panel Studies and Peking University Digital Financial Inclusion Index Database to conduct an empirical investigation. The results provided empirical evidence of the positive effect of a major public health emergency outbreak on individual commercial insurance, commercial endowment insurance, and commercial medical insurance. The main conclusions of this study are as follows: (1) The COVID-19 outbreak can significantly improve the individual participation probability of commercial insurance, endowment insurance, and commercial medical insurance, and the result is robust after a series of tests. (2) There are at least three mechanisms for the positive impact of the COVID-19 outbreak: increasing individual risk perception, individual risk preference and digital finance. (3) The positive impact of the COVID-19 outbreak on commercial medical insurance was more prominent among urban residences, high-income populations and women. In addition, there is a “neighborhood effect” in commercial insurance decision-making.

This study makes the following policy recommendations to minimize the impact of severe health emergencies on the commercial insurance market. First, insurance institutions should improve their products according to the characteristics of public health emergencies. Innovative digital insurance is a beneficial supplement to traditional insurance marketing practices. With the help of advanced technologies such as the Internet, big data and artificial intelligence, insurance institutions can strengthen cooperation and publicity with e-commerce platforms, social media and other online channels to promote insurance awareness and demand, risk cognition and long-term risk management of individuals. Second, the impact of the COVID-19 outbreak on the commercial insurance participation of vulnerable groups was relatively limited. Therefore, financial institutions should tap into potential customers by focusing on vulnerable societal groups by optimizing the pricing strategy of insurance products, designing more targeted and diversified insurance products and providing convenient insurance purchase options for them. Moreover, financial institutions should fully leverage the neighborhood effect of insurance participation and drive the participation of vulnerable groups in commercial insurance. Third, the government should formulate digital financial ability training policies based on the specific characteristics of groups and promote the orderly development and popularization of financial ability education.

## Data availability statement

The raw data supporting the conclusions of this article will be made available by the authors, without undue reservation.

## Author contributions

YW: Conceptualization, Resources, Writing—review & editing. CG: Conceptualization, Data curation, Formal analysis, Writing—original draft, Methodology, Visualization. YX: Methodology, Software, Writing—review & editing. MX: Investigation, Supervision, Writing—review & editing.

## Footnotes

^1^The number of COVID-19 cases in Shanghai in September exceeded 1,000. Since the interview time in the questionnaire only lists the month, we have excluded samples whose interview date is after August 2020.
